# Comparison of nicardipine versus labetalol for time to alteplase administration in acute ischemic stroke

**DOI:** 10.3389/fneur.2025.1573352

**Published:** 2025-07-02

**Authors:** Alex Huang, Dennis Parker, Rachel Wein

**Affiliations:** ^1^Department of Pharmacy, Detroit Receiving Hospital, Detroit, MI, United States; ^2^Eugene Applebaum College of Pharmacy and Health Sciences, Wayne State University, Detroit, MI, United States

**Keywords:** alteplase, ischemic stroke, administration time, door to needle time, nicardipine, labetalol

## Abstract

**Background:**

Though the acute management of hypertension in patients with ischemic stroke has been associated with prolonged time to alteplase administration, it is unclear whether the choice of antihypertensive agent also influences this outcome. The purpose of this study was to evaluate the difference between nicardipine and labetalol on door-to-needle time in hypertensive patients with acute ischemic stroke.

**Methods:**

This multicenter, retrospective study included all adult patients presenting to the emergency department (ED) with acute ischemic stroke between January 2018 and August 2023 who received at least one dose of labetalol or nicardipine prior to thrombolytic therapy. Patients with concomitant acute myocardial infarction, bradycardia, or history of severe aortic stenosis were excluded from the analysis. The primary outcome of this study was the time from ED arrival to alteplase administration.

**Results:**

A total of 481 patients were included in the analysis: 400 (83%) received no antihypertensive, 68 (14%) received labetalol, and 13 (3%) received nicardipine prior to alteplase administration. We found no statistically significant difference in median door-to-needle times in patients who received labetalol vs. those that received nicardipine (63 min vs. 81 min, *p* = 0.19). Patients who did not require antihypertensive treatment had a shorter median door-to-needle time than those treated with either labetalol or nicardipine (58 min vs. 67 min, *p* = 0.02).

**Conclusion:**

This study did not demonstrate a significant difference in time to alteplase administration between patients treated with labetalol vs. nicardipine. Further studies are needed to determine whether the choice of antihypertensive agent used in acute ischemic stroke significantly affects clinical outcomes.

## Introduction

Though the decision to initiate thrombolytic therapy in patients with acute ischemic stroke is influenced by several factors, the benefit of intravenous (IV) alteplase remains time dependent. Previous studies have demonstrated that for every 15-min reduction in door-to-needle time, there is a significantly reduced risk of in-hospital mortality, all-cause mortality, and hospital readmission (1, 2). For this reason, current AHA/ASA guidelines recommend a primary goal of achieving door-to-needle times within 60 min in at least 50% of acute ischemic stroke patients treated with IV alteplase (3, 4).

The acute management of hypertension in patients with ischemic stroke represents a potentially modifiable delay in door-to-needle time. Based on current AHA/ASA guidelines, blood pressure should be lowered to less than 185/110 mmHg prior to IV alteplase and maintained at less than 180/105 for at least 24 h after fibrinolytic treatment. Elevated pre-hospital blood pressures and acute blood pressure management have been associated with significant delays in door-to-needle time, with approximately 20% of patients with acute ischemic stroke requiring antihypertensive therapy prior to treatment with alteplase (5–7). However, the optimal approach to blood pressure management in acute ischemic stroke is still unknown, as current consensus guideline recommendations for first-line agents (nicardipine, labetalol, clevidipine) and alternatives (hydralazine, enalaprilat) are based on expert opinion (3).

**Figure 1:**
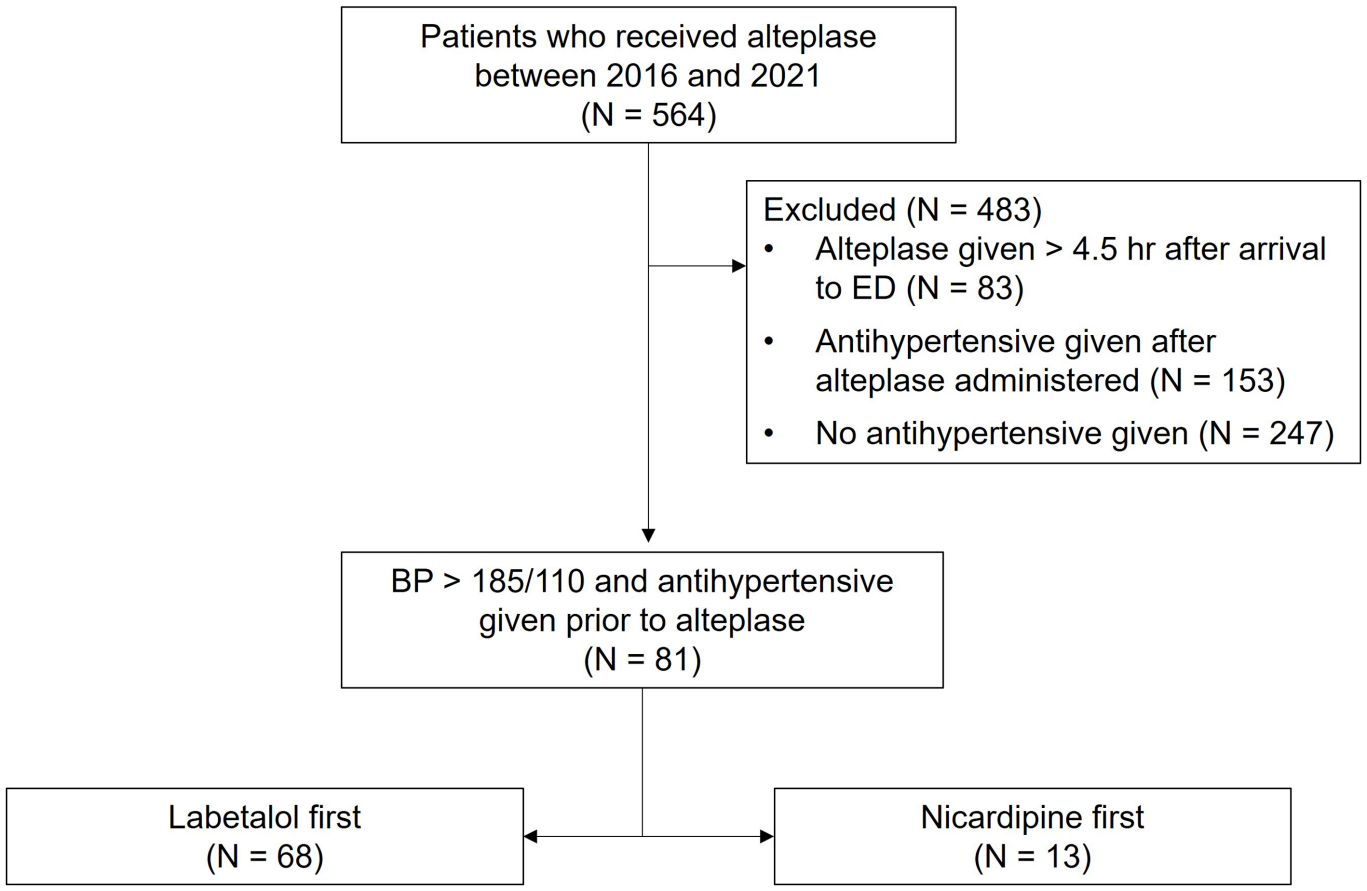
Study population.

A limited amount of clinical data is available to inform antihypertensive selection for patients with acute ischemic stroke. Data from retrospective and prospective studies in acute stroke suggest that nicardipine-treated patients are more likely to reach their target BP within 30 min, experience less blood pressure variability and remain within the goal blood pressure range longer compared to patients treated with labetalol (8, 9). However, these studies did not demonstrate a significant difference in clinical outcomes (e.g., adverse effects, in-hospital mortality, ICU length of stay) between nicardipine and labetalol groups. Furthermore, patients with acute ischemic stroke only represented about one-third of the cohort across both studies (see Figure 1).

While nicardipine has been shown to produce a faster and more controlled blood pressure response compared to labetalol in acute stroke, it is unclear whether the theoretically advantageous blood pressure reduction profile of nicardipine influences more clinically oriented outcomes such as door-to-needle time. Given these uncertainties, the purpose of this study was to evaluate the difference between nicardipine and labetalol on door-to-needle time in hypertensive patients with acute ischemic stroke.

## Methods

This is a retrospective chart review of patients with acute ischemic stroke who were admitted to one of two university-affiliated, urban medical centers in the Detroit metropolitan area between 2018 and 2023. Both sites are Joint Commission certified Advanced Primary Stroke Centers and maintain stroke code response teams comprised of a neurology fellow and on-call attending physician, an ED resident and attending physician, ED nurses, and an ED pharmacist. We included patients who received alteplase within 4.5 h of symptom onset, had a measured blood pressure of greater than 185/110 mmHg prior to alteplase administration, and received at least one dose of labetalol or nicardipine. Antihypertensive selection was at the discretion of the treating team and patients were assigned to the labetalol or nicardipine group based on the initial agent administered. Those who did not receive any antihypertensive agent prior to alteplase were analyzed separately. Patients were excluded if they presented with concomitant acute myocardial infarction, bradycardia (heart rate <60 bpm), or had a history of severe aortic stenosis. Study data including patient demographics, pre-existing diagnoses, time of arrival, blood pressures, and medication administration information (alteplase and antihypertensive dose, dose titrations, and timing) were directly obtained from the electronic medical record. Patient charts were manually reviewed to confirm the National Institute of Health Stroke Scale score (NIHSS) on arrival and differentiate between symptomatic and asymptomatic hemorrhagic transformation. Symptomatic ICH (sICH) was defined as type 2 parenchymal hemorrhage (PH2) accompanied by neurological deterioration.

The primary outcome of this study was the door-to-needle time in minutes, defined as the time from ED arrival to alteplase administration. Secondary efficacy outcomes included order-to-needle time (time in minutes from alteplase order verification to administration), antihypertensive-to-needle times (time in minutes from the first dose of antihypertensive to alteplase administration), time to blood pressure control (time in minutes from the first dose of antihypertensive to blood pressure <185/110 mmHg) and need for additional antihypertensives (the addition of nicardipine to labetalol, or vice versa). Safety outcomes included the incidence of symptomatic hemorrhagic transformation, and incidence of hypotension (defined as systolic blood pressure <90 mmHg) or bradycardia (defined as heart rate <60 bpm). Study groups were compared using the Mann–Whitney *U* test for continuous data or Pearson’s chi-square test for categorical data using R version 4.1.3 (R Core Team, Vienna, Austria). *p*-values <0.05 were considered statistically significant for all tests. This study was approved by the Institutional Review Board of Wayne State University with a waiver of informed consent.

## Results

A total of 481 patients were included in the analysis: 400 (83%) received no antihypertensive, 68 (14%) received labetalol, and 13 (3%) received nicardipine prior to alteplase administration. Patients in the nicardipine group had higher initial NIHSS scores compared to those in the labetalol group (median score 16 vs. 8, *p* = 0.001). Baseline characteristics were otherwise similar between the nicardipine and labetalol groups (Table 1).

**Table 1:** Baseline characteristics.

Characteristic	Labetalol (*N* = 68)	Nicardipine (*N* = 13)	*p*-value
Age, years	65 (58–76)	69 (66–85)	0.12
Male sex	31 (46%)	6 (46%)	0.97
Race and ethnicity			
Black/African American	49 (72%)	8 (62%)	0.45
White	5 (7%)	0 (0%)	0.31
Unknown/Other^a^	14 (21%)	5 (38%)	0.16
Vascular risk factors			
Hypertension	40 (60%)	11 (85%)	0.08
Afib/Aflutter	10 (15%)	2 (15%)	0.95
Diabetes	28 (41%)	8 (62%)	0.18
History of CAD/MI	19 (28%)	4 (31%)	0.84
Dyslipidemia	30 (44%)	6 (46%)	0.89
History of stroke/TIA	17 (25%)	3 (23%)	0.88
Heart failure	18 (26%)	6 (46%)	0.15
NIHSS score	8 (5–13)	16 (10–20)	0.001
0–4 (mild)	12 (18%)	0 (0%)	
5–15 (moderate)	44 (65%)	7 (44%)	
16–20 (mod-severe)	8 (12%)	6 (38%)	
>20 (severe)	4 (6%)	3 (19%)	
Peak SBP prior to alteplase, mmHg	190 (176–207)	186 (182–199)	0.65
Peak DBP prior to alteplase, mmHg	114 (104–123)	107 (96–113)	0.13

Values reported as median (IQR) or *n*(%).

a Includes Asian, American Indian/Alaskan Native, Native Hawaiian/Pacific Islander, mixed race, or any other nonblack or nonwhite race categories.

In terms of antihypertensive management, 63% of patients in the labetalol group received an initial dose of 20 mg and 37% received an initial dose of 10 mg. 57% of patients in the labetalol group only required a single dose of labetalol for blood pressure management prior to alteplase. Most patients in the nicardipine group (92%) were started at an initial rate of 5 mg/h, with one patient started at an initial rate of 2.5 mg/h. 77% of patients in the nicardipine group required no further dose titration beyond the initial rate. Further details regarding the antihypertensive dosing regimens utilized in this study are provided in Table 2.

**Table 2:** Antihypertensive dosing regimens.

Dosing	Labetalol (*N* = 68)	Nicardipine (*N* = 13)
Initial dose, mg or mg/h		
Median (IQR)	20 (10–20)	5.0 (5–5)
Mean (SD)	16 (5.0)	4.8 (0.7)
Dose prior to alteplase, mg or mg/h^a^		
Median (IQR)	20 (15–40)	5.0 (3–5)
Mean (SD)	25 (14.1)	5.6 (3.8)
Doses administered/titrated^b^		
1	39 (57%)	10 (77%)
2	24 (35%)	—
3+	5 (7%)	3 (23%)

a Cumulative dose of labetalol or drip rate of nicardipine immediately prior to alteplase.

b For patients in the labetalol group, each dosing category corresponds to total the number of doses administered prior to alteplase. For patients in the nicardipine group, each dosing category corresponds to the total number of dose titrations (defined as an incremental change in rate of 2.5 mg/h) prior to alteplase inclusive of the initial starting rate. Thus, patients in category 1 are those who did not require any additional changes to the initially ordered nicardipine drip rate, patients in category 2 required one dose titration, and patients in category 3+ required two or more dose titrations prior to alteplase.

We found no statistically significant difference in the primary outcome of median door-to-needle time in patients who received labetalol vs. nicardipine prior to alteplase (63 vs. 81 min, *p* = 0.19). Median order-to-needle times (16 vs. 27 min, *p* = 0.52), antihypertensive-to-needle time (31 vs. 32 min, *p* = 0.94), time to blood pressure control (20 vs. 26 min, *p* = 0.89) and the proportion of patients requiring the addition of a second antihypertensive agent (32% vs. 38%, *p* = 0.59) were also similar between labetalol and nicardipine groups (Table 3). Patients who did not require antihypertensive treatment had a shorter median door-to-needle time compared to patients treated either labetalol or nicardipine (58 vs. 66 min, *p* = 0.03). The incidence of bradycardia, hypotension, and symptomatic intracerebral hemorrhage was similar between the labetalol and nicardipine groups (Table 4).

**Table 3:** Primary and secondary efficacy endpoints.

Endpoint	Labetalol (*N* = 68)	Nicardipine (*N* = 13)	*p*-value
Door-to-needle time, min	63 (50–86)	81 (58–104)	0.19
Order-to-needle time, min	16 (10–31)	27 (6–46)	0.52
Antihypertensive-to-needle time, min	31 (19–53)	32 (19–45)	0.94
Need for additional antihypertensive^a^	21 (32%)	5 (38%)	0.59

Values reported as median (IQR).

a Specifically, the addition of nicardipine to labetalol, or labetalol to nicardipine.

**Table 4:** Adverse events.

Adverse event	Labetalol (*N* = 68)	Nicardipine (*N* = 13)	*p*-value
Bradycardia (HR <60)	13 (19%)	2 (15%)	0.10
Hypotension (SBP <90)	3 (4%)	0 (0%)	0.44
Symptomatic intracerebral hemorrhage	1 (1%)	1 (8%)	0.19
In-hospital mortality	2 (2.9)	2 (15.4)	0.058
30-day mortality	3 (4.4)	2 (15.4)	0.132

Values reported as *n*(%).

## Discussion

In this retrospective, multicenter study of patients with acute ischemic stroke requiring antihypertensive therapy prior to alteplase, we found no statistically significant difference in the primary outcome of median door-to-needle time between labetalol or nicardipine groups. Additionally, no statistically significant differences in secondary efficacy outcomes including median order-to-needle time, antihypertensive-to-needle time, time to blood pressure control, and need for an additional antihypertensive agent were observed between labetalol and nicardipine groups. The incidence of adverse effects was similar between both groups.

Our study findings add to the limited amount of data regarding the optimal management of blood pressure in patients with acute ischemic stroke. To date, there have been three retrospective studies comparing door-to-needle times across antihypertensive agents. McKay et al. (10) included 25 patients treated with labetalol, 3 with nicardipine, and 1 with hydralazine and found median door-to-needle times of 74 min (IQR 39–218), 91 min (IQR 75–112), and 34 min, respectively. Due to limitations of sample size, the authors did not report any statistical comparison between the groups. Carrera et al. (11) published a single-center, retrospective study of 239 patients with acute ischemic stroke: 177 who received no antihypertensive, 44 labetalol, 13 nicardipine, and 5 hydralazine. They found no statistically significant difference in mean door-to-needle times across the three agents (labetalol 64.3 min, nicardipine 53.0 min, hydralazine 67.4 min, *p* = 0.052). Finally, the most recent of these studies by Kamp et al. (12) included 122 patients who received an intermittent antihypertensive (105 labetalol, 17 hydralazine) and 57 patients who received a continuous infusion antihypertensive (29 nicardipine, 28 clevidipine) in a multicenter, retrospective, cohort study. They found no statistically significant difference in median door-to-needle times between the intermittent and continuous infusion groups (53 vs. 57 min, *p* = 0.17).

Antihypertensive selection may influence door-to-needle time if blood pressure control can be achieved more rapidly with one agent compared to another. In this study, we found no significant difference in secondary endpoints related to acute blood pressure management between labetalol and nicardipine groups, including the antihypertensive-to-needle time (31 vs. 32 min, *p* = 0.94), time to blood pressure control (20 vs. 26 min, *p* = 0.89) and the proportion of patients requiring the addition of a second antihypertensive agent (32% vs. 38%, *p* = 0.59). These data contrast with previously published literature which suggested that patients with acute stroke who receive nicardipine are more likely to reach their target BP within 30 min compared to patients treated with labetalol (8, 9). However, these studies primarily included patients with acute hemorrhagic stroke, for whom typical blood pressure targets differ significantly compared to those with acute ischemic stroke eligible for thrombolytic therapy (SBP <140–160 mmHg vs. BP <185/110 mmHg, respectively). Instead, our study findings align with Kamp et al. (12), who also found no significant difference in the time from initial antihypertensive administration to BP target <185/110 mmHg in patients receiving an intermittent vs. continuous infusion antihypertensive. Taken together, these data suggest that labetalol and nicardipine provide similarly rapid control of blood pressure prior to alteplase administration.

While our study may have been underpowered to detect a difference in door-to-needle time between labetalol and nicardipine groups due to its small sample size, the modest but statistically significant difference in door-to-needle times between antihypertensive-treated and no antihypertensive groups observed in this study suggests that any potential difference due to the antihypertensive agent used may be even smaller. The size of the delay in door-to-needle time observed in this study between antihypertensive-treated and non-antihypertensive-treated patients is consistent with published literature. A secondary analysis of the INSTINCT trial found patients requiring pre-thrombolytic antihypertensive treatment had a mean increase in door-to-needle time of 9 min (95% confidence interval, 2–16 min) compared to than those that did not (7). Carrera et al. (11) observed a 9.5-min delay in door-to-needle time associated with patients who required antihypertensive therapy prior to alteplase treatment (62.1 min versus 52.6 min, *p* = 0.02). In the present study, we found that patients who required antihypertensive treatment experienced a median increase in door-to-needle time of 8 min compared to those that did not (66 vs. 58 min, *p* = 0.03). If acute blood pressure management can delay alteplase administration, it stands to reason that any potential benefit on door-to-needle time derived from optimal antihypertensive selection is unlikely to exceed the absolute difference in door-to-needle time observed between patients who require antihypertensives and those who do not. Given that the expected magnitude of the effect size (i.e., difference in door-to-needle time) is relatively small, the numerical difference in median door-to-needle times between labetalol and nicardipine groups (63 vs. 81 min, *p* = 0.19) observed in this study is likely due to factors unrelated to differences in blood pressure reduction profiles between the two agents and is not statistically significant.

Our study has several notable limitations. First, due to its retrospective design we were unable to control for certain confounding factors (e.g., time spent determining alteplase eligibility, time spent in CT scanner) and other sources of bias that may also have affected the primary outcome. Secondly, there was an imbalance in the number of patients who received labetalol and nicardipine and a small number of patients overall. Based on the prescribing patterns observed in similar studies, while the treatment teams’ preference for labetalol is reflective of a real-world tendency to favor intermittent bolus therapy over continuous infusions in the management of hypertensive patients with acute stroke, having a larger sample of patients who received nicardipine initially would have helped to better characterize door-to-needle times in that group. Finally, it is unclear to what extent the higher baseline stroke severity in the nicardipine group may have affected the primary outcome. While a strong association between NIHSS score and door-to-needle time has not been established in the literature (1), higher stroke severity may have influenced overall treatment decisions and initial antihypertensive selection, indirectly affecting door-to-needle times.

## Conclusion

Though nicardipine has previously been shown to achieve faster blood pressure control compared to labetalol in patients with acute stroke, this study did not demonstrate a significant difference in time to alteplase between patients treated with labetalol vs. nicardipine. Consistent with previously published studies, median door-to-needle times were shorter in patients who did not require antihypertensive treatment compared to those who received labetalol or nicardipine prior to alteplase administration. Further studies are needed to determine whether the choice of antihypertensive agent used in acute ischemic stroke significantly affects clinical outcomes.

## Data Availability

The raw data supporting the conclusions of this article will be made available by the authors, without undue reservation.
